# Relationships of gaming disorder, ADHD, and academic performance in university students: A mediation analysis

**DOI:** 10.1371/journal.pone.0300680

**Published:** 2024-04-03

**Authors:** Nazir Hawi, Maya Samaha

**Affiliations:** 1 Notre Dame University-Louaize, Zouk Mosbeh, Lebanon; 2 Institute of Internet and Technology Addiction, Notre Dame University-Loauize, Zouk Mosbeh, Lebanon; University of Connecticut Health Center: UConn Health, UNITED STATES

## Abstract

This study investigates the intersection of Gaming Disorder (GD) and Attention-Deficit/Hyperactivity Disorder (ADHD), and Grade Point Average (GPA), among university students, a critical demographic often overlooked in research on these disorders. A sample of 348 university students was analyzed using the IGD-20 Test for risk of GD, the Adult ADHD Self-Report Scale (ASRS-v1.1) for ADHD symptoms, and GPA as a metric of academic performance. The findings indicate that 4.3% of the surveyed sample scored within the range for GD. The prevalence was higher in males, with 5.3% of the male cohort affected, compared to 1.2% of the female cohort. Significantly, the prevalence of ADHD was substantially higher in the GD group (35.7%) than in the non-GD group (24.2%). Further, ADHD symptoms were found to be a stronger predictor of GD in females than in males. Incorporating the mediating role of Gaming Disorder, this study also probes into how GD may serve as an intermediary in the impact of ADHD on academic performance. By examining the intricate relationship between these disorders, our findings suggest that GD exacerbates the negative effects of ADHD on academic performance, thereby underscoring the potential for Gaming Disorder to act as a bridge in this dynamic. This mediation analysis clarifies how ADHD may indirectly impact academic performance through GD. The study reveals a positive correlation between ADHD symptoms and GD severity, which in turn correlates negatively with academic achievement. In addition, the findings underscore the need for gender-sensitive interventions and highlight the importance of considering the comorbidity of ADHD and GD in academic settings, advocating for systematic screening for GD among students with ADHD, and vice versa. The dual challenges posed by ADHD and GD should be addressed to prevent their escalation into pervasive academic and psychosocial adversities.

## Introduction

While the world is witnessing a major evolution in technology and artificial intelligence, new related challenges have emerged, particularly related to mental disorders [1–6]. For instance, Internet Gaming Disorder (IGD) was included in the DSM-5 [7] and is characterized by excessive and compulsive engagement in internet games, significantly disrupting personal, familial, social, and occupational domains. In 2018, the World Health Organization (WHO) recognized Gaming Disorder (GD) in the International Classification of Diseases (ICD-11) as a pattern of behavior involving persistent engagement in video games, often characterized by an inability to control playing time and a prioritization of gaming over other life interests and daily activities. This behavior persists or escalates despite negative consequences, disrupting personal, family, social, educational, or occupational functioning [8].

Attention-Deficit/Hyperactivity Disorder (ADHD), on the other hand, is typified by persistent inattention, hyperactivity, and impulsivity that substantially impair functioning or development. Recent research has sought to understand the potential associations between GD and ADHD. A systematic review and meta-analysis indicated that individuals with ADHD have significantly higher GD severity, and vice versa, suggesting the necessity for ADHD screening in individuals with GD and raising awareness of GD risks in ADHD individuals [9]. Other studies have highlighted a moderate to strong comorbidity between GD and ADHD, with the strength of this relationship influenced by factors such as gender, type of internet use, and assessment methods [10, 11].

This relationship is further complicated by findings that associate a higher severity of GD in ADHD individuals to a range of mental health disorders, including OCD, anxiety, and depression [12–14]. Additional research indicates that specific types of games and gaming behaviors, such as action or immersive games, have varying impacts on addiction levels and gaming characteristics in those with ADHD [15–17]. The interplay of these disorders also extends to lifestyle aspects, with technology overuse affecting sleep and daytime sleepiness in ADHD adolescents [18], and gaming being identified as a potential coping mechanism for emotional dysregulation in ADHD population [19]. This complexity is further deepened by the mediating role of depression symptoms in GD [20].

Meanwhile, studies have expanded this understanding to diverse cultural populations, revealing the prevalence of GD and its correlation with ADHD symptoms [21], particularly inattention, and emphasizing the need for symptom-focused research in GD [22–24]. Overall, the literature review exposes a multifaceted and complex relationship between GD and ADHD, underscoring the need for further nuanced and targeted research in this field. Additionally, several studies explored the negative relationships between GD and academic performance in university settings [25–31]. However, the nuanced relationship between GD, ADHD, and academic performance among university students remains underexplored, with limited data on how they correlate and differ across genders in university student populations. This research aims to study the complex interplay between GD and ADHD within this context, providing an in-depth analysis of their prevalence, intersection, and relation with academic performance. Herein, are the research questions:

- Is there a significant difference in ADHD symptom severity between individuals with and without Gaming Disorder?
- Are there gender differences in the prevalence and severity of GD and ADHD symptoms?
- How do GD and ADHD symptoms relate to academic performance among university students?
- Does GD have a mediating role between ADHD symptoms and academic performance?

Given the considerable prevalence rates of GD and ADHD found in preliminary studies and the significant academic stakes, there is a pressing need to understand these conditions’ interrelation. Such insights are important for developing tailored intervention strategies to support affected students.

## Materials and methods

### Procedure and sample

This study utilized a voluntary, cross-sectional approach to investigate the relationships between GD, ADHD, and GPA, among university students in Lebanon. The research was approved by the university’s Institutional Research Board ensuring compliance with ethical standards. We adopted convenience sampling as our technique for participants selection. Participants were recruited through university mailing lists and social media platforms between first of June and end of July 2019. Before taking the online survey, participants were provided with a section detailing the study’s purpose and guaranteeing that all data collection, storage, and reporting methods would uphold their confidentiality and anonymity. After providing their electronic informed consent (econsent), participants were given access to the survey for completion. A total of 383 respondents filled out the survey. Cases with invalid responses to the trap question were excluded from the analysis, which reduced the sample size to 348 (*M*_*age*_ = 19.93, *SD* = 2.08). The ages of respondents ranged between 17 and 26 years. Of the 348 valid cases, 73.9% were males. The higher proportion of males in the sample could stem from cultural biases that make gaming more socially accepted for males than females, thereby influencing their participation rate in the study. Additionally, males might have felt a stronger personal connection or perceived the topic as more relevant to their experiences, leading to a higher inclination to participate.

### Instruments

The survey, administered in the English language, was composed of three separate sections, including one for demographic information and two for separate research instruments. The demographic information section included age, gender, average number of hours spent on gaming per day on weekdays as well as other days including weekends and holidays, and GPA. The remaining sections encompassed both the IGD-20 Test and the Adult ADHD Self-Report Scale (ASRSv1.1). Instructions were clearly stated, emphasizing the anonymity and confidentiality of their responses. The amount of time required to complete the survey was approximately 20 minutes.

To assess Gaming Disorder, the IGD-20 Test was utilized [32]. This self-report instrument is comprised of 20 items that align with the DSM-5 criteria for Gaming Disorder (GD) and incorporate the theoretical framework of the components model of addiction [33]. The test examines online and offline gaming activities occurring over a 12-month period and focuses on persistent and recurrent gaming behavior. Items are rated on a five-point Likert scale: 1 "Strongly disagree," 2 "Disagree," 3 "Neither agree or disagree," 4 "Agree," and 5 "Strongly agree." Responses are summed to determine the presence of gaming disorder, with a cut-off score of 71, as recommended by Pontes et al. [32]. Scores at or above this threshold indicate the presence of gaming disorder. The mean score for the IGD-20 Test was 41.83 (*SD* = 14.35). The IGD-20 Test has demonstrated good psychometric properties in previous studies [34]. The IGD-20 Test demonstrated strong internal consistency in this study, with a Cronbach’s Alpha of.87.

ADHD symptoms were assessed using the Adult ADHD Self-Report Scale (ASRS v1.1), which is a self-assessment tool designed to help screen for adult attention-deficit/hyperactivity disorder (ADHD). Created by the World Health Organization (WHO), the ASRS v1.1 is based on the criteria set forth in the American Psychiatric Association’s Diagnostic and Statistical Manual of Mental Disorders (DSM-IV). The ASRS v1.1 consists of two parts. Part A has six questions that are designed to screen for adult ADHD. These are derived from the most predictive symptoms of ADHD. Part B has the remaining 12 questions that help further probe the symptoms of ADHD. The primary goal of the ASRS v1.1 is to provide a standardized, consistent, and reliable tool to help identify adults who may need further assessment for ADHD. It’s important to note that the scale itself is not intended to diagnose ADHD but to be a first step in the screening process. The ADHD scale showed strong internal consistency, with a Cronbach’s Alpha of.87, corroborating the reliability of the measure.

Stanton et al. (2018) identified two subscales of ASRS v1.1 [35]:

The Inattentive subscale evaluates an adult’s difficulties in areas such as detail focus, organization, remembering appointments, avoiding careless mistakes, and sustaining concentration. It encompasses items 1, 2, 3, 4, 7, 8, 9, 10, and 11, the scoring scale ranges from 0 to 9.

The Hyperactive/Impulsive subscale evaluates an adult’s motor challenges with items 5, 6, 12, 13, and 14, and verbal difficulties with items 15, 16, 17, and 18, with the scoring scale ranging from 0 to 9.

The scoring of these ASRS v1.1 subscales is as follows: for items 1–3, 9, 12, 16, and 18, a score of one point is given for responses of "sometimes," "often," or "very often," while responses of "never" or "rarely" receive zero points. For the other 11 items, a score of one point is awarded for "often" or "very often" responses, with "never," "rarely," or "sometimes" responses receiving zero points.

### Statistical analysis

Data were analyzed using SPSS Version 25. Descriptive statistics were calculated for demographic variables and scale scores. Pearson’s correlation coefficient was employed to examine the relationship between GD, ADHD, and GPA. Regarding the assumptions for the Pearson’s correlation analysis, linearity was examined by inspecting scatterplots of the research variables. The scatterplots displayed linear relationships. Homoscedasticity was also assessed through visual inspection of these scatterplots, which revealed a uniform spread of residuals across the range of predicted values, suggesting that the variance of the error term is constant across values of the independent variables in question. Prior to conducting the independent-samples T-Test, we verified the assumption of normality and homogeneity of variances. Normality was assessed using the Shapiro-Wilk test for each group, which did not indicate significant deviations from normality (p >.05 for all groups). Homogeneity of variances was tested using Levene’s Test for Equality of Variances. The results were non-significant (p >.05), indicating no substantial difference in variances between the groups being compared, which satisfies the assumption of equal variances required for the independent-samples T-Test. Further, regression analyses were conducted to explore the predictive value of ADHD symptoms for GD.

A mediation analysis was conducted using structural equation modeling (SEM) with IBM SPSS AMOS Version 24 to test the potential mediating role of GD in the relationship between ADHD symptoms and academic performance.

## Results

### Descriptive statistics

In our study sample of size 348 respondents, the prevalence of GD was 4.3%. The mean age for participants with disordered gaming (Disordered Gaming Group, DGG) was 20.6 years (*SD* = 1.75), with 92.9% being male (Table 1). The mean age for both the ADHD group and the non-ADHD group was 19.9 years. The prevalence of GD was 6.5% in the ADHD group and 3.7% in the non-ADHD group. The mean age was 20.1 years for the DGG group and 19.4 years for the non-DGG group. Among these, 35.7% of the DGG group and 23.3% of the non-DGG group exhibited symptoms of ADHD. Gender differences in GD prevalence were 5.3% for males and 1.2% for females. Within the ADHD cohort, 24.6% of males and 22.1% of females exhibited symptoms of ADHD. An independent-samples T-Test showed no significant difference in ADHD scores between males (*M* = 24.6, *SD* = 0.4) and females (*M* = 22.1, *SD* = 0.4; *t*(346) = 0.5, *p = 0*.642).

**Table 1:** Characteristics of the study sample.

Group	Prevalence Rate within Sample	Mean Age	Gender Distribution (male %)
DGG	4.3%	20.6	92.9%
ADHD Overall	24.2%	19.9	75.9%
Good AP*	27.3%	19.5	60%
DGG (within Males)	5.4%	20.4	100%
DGG (within Females)	1.2%	23.0	0%
ADHD in DGG	35.7%	19.6	80.0%
ADHD in non-DGG	23.3%	19.8	76.4%
Good AP* in DGG	8.3%	21.0	100%
Good AP* in non-DGG	100%	19.9	73.5%

Note.

*AP = Academic Performance

A Pearson’s correlation analysis revealed a moderate positive relationship between GD and average gaming hours on university days (*r* = .365, *p <* .001), and a strong positive relationship on non-university days (*r* = .487, *p <* .001). A small negative relationship was found between IGD and average sleep hours (*r = -*.151, *p <* .001). The DGG averaged 3.6 hours (*SD* = 2.7) of gaming on university days and 7.7 hours (*SD* = 4.8) on non-university days. For the non-DGG, females averaged fewer gaming hours than males, with 1.4 (*SD* = 2.1) hours on university days and 2.2 (*SD* = 2.2) hours on non-university days for females, compared to 2.0 (*SD* = 2.2) hours and 3.8 (*SD* = 3.2) hours for males, respectively.

For the ADHD group, there was no difference in average gaming hours between genders on university days, with both averaging 2.1 hours, though males played more on non-university days, averaging 3.8 hours (*SD* = 3.2) compared to 2.2 hours (*SD* = 2.2) for females. In the non-ADHD group, females averaged 1.2 hours (*SD* = 2.0) and males 2.0 hours (*SD* = 2.3) on university days, with non-university day averages at 2.1 hours (*SD* = 2.0) for females and 3.9 hours (*SD* = 3.4) for males.

The DGG’s average sleep duration was 6.1 hours (*SD* = 1.3), with 64.3% experiencing nocturnal awakenings to game. In contrast, the non-DGG had higher sleep averages of 7.2 hours (*SD* = 2.1) for females and 6.8 hours (*SD* = 1.9) for males, with nocturnal awakenings being negligible in minutes count for males and absent for females.

### Correlation analysis

A Pearson’s correlation analysis revealed a significant positive relationship between GD and ADHD scores, *r* = .33, *p <* .001 (Table 2). This correlation suggests that higher levels of ADHD symptoms are associated with higher levels of IGD symptoms. Within the female’s cohort, the Pearson’s correlation showed a strong positive relationship between IGD and ADHD scores, *r* = .49, *p <* .*001*, while it was moderate, *r* = .21, *p <* .001, for male’s cohort.

**Table 2:** Correlation analysis of IGD, ADHD, and academic performance.

Variable Comparison	*r*	*p*	Notes
IGD and ADHD Scores (Overall)	0.33	< .001	Positive correlation suggests higher ADHD symptoms associated with higher IGD levels.
IGD and ADHD Scores (Females)	0.49	< .001	Strong positive correlation; however, based on a very small sample size (n = 1 for DGG females).
IGD and ADHD Scores (Males)	0.21	< .001	Moderate positive correlation.
IGD and Academic Performance	-0.18	< .001	Moderate negative correlation; higher IGD levels associated with lower academic performance.
ADHD and Academic Performance	-0.18	< .001	Moderate negative correlation; higher ADHD levels associated with lower academic performance.

**Fig 1:**
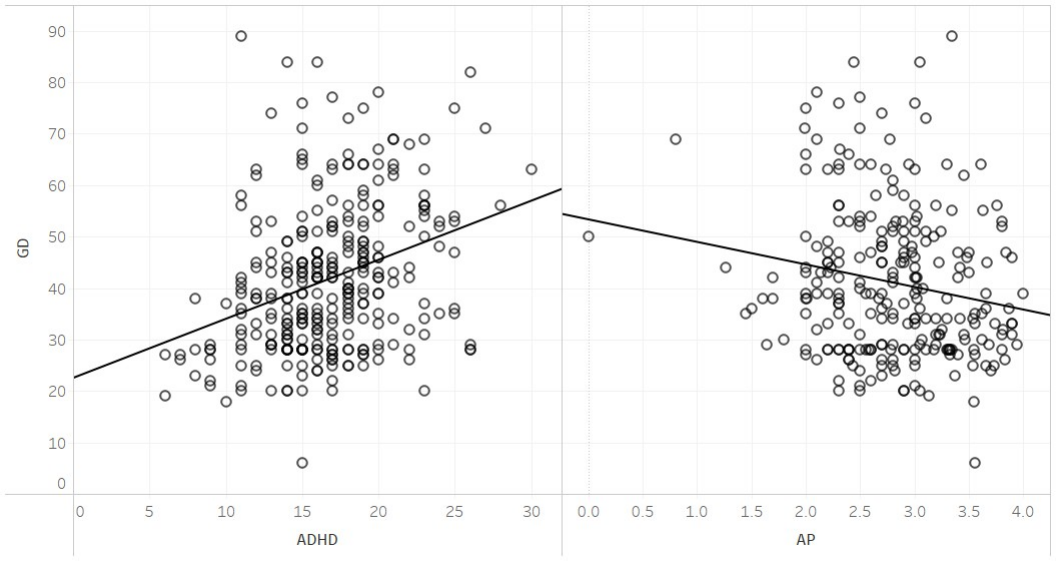
Correlations between GD, and ADHD and GPA.

It is noteworthy that 35.7% of DGG met the criteria for ADHD, while 24.2% of the entire sample met the criteria (Table 2). Within the DGG, 30.8% of males met the criteria for ADHD. The majority of respondents in the Disordered Gaming Group (DGG) were males. The group included only one female, who met the criteria for ADHD. While this finding is not indicative due to the small female sample size in the DGG, it warrants further investigation to understand the relationship between gender, gaming disorder, and ADHD. While 93.5% of the respondents who met the criteria of ADHD showed symptoms of GD, only 6.5% did not show symptoms of GD (Fig 1).

At the sample’s level, a Pearson’s correlation analysis revealed a moderate negative relationship between GD and ADHD on one hand and the academic performance excellence, *r = -*.18, *p <* .001, and *r = -*.18, *p <* .001, respectively. Among the respondents classified as high academic performers, with GPAs of 3.2 and above, only one met the criteria for symptoms of GD or ADHD.

A Pearson’s correlation analysis was conducted to examine the relationship between IGD scores and the ASRS v1.1 subscales scores, namely Inattention and Hyperactivity/Impulsivity scores. The analysis revealed a significant positive relationship between GD and Inattention scores, *r* = .31, *p* < .001. This correlation suggests that higher levels of Inattention symptoms are associated with higher levels of IGD symptoms. Within the female’s cohort, the Pearson’s correlation showed a strong positive relationship between IGD and Inattention scores, *r* = .41, *p* < .001, while it was moderate, *r* = .30, *p* < .001, for male’s cohort. Also, the analysis revealed a significant positive correlation between GD and Hyperactive/Impulsive scores, *r* = .24, *p* < .001, indicating that higher levels of IGD are associated with increased Hyperactive/Impulsive symptoms. Moreover, across both subscales, there is a notable variability in symptom severity among the DGG. This indicates a diverse profile of ADHD symptomatology within the group, with some individuals showing minimal symptoms and others experiencing more severe manifestations.

### Regression analysis

To further explore the predictive value of ADHD for IGD, linear regression analysis was conducted. The ADHD scale score was a significant predictor of IGD, *β* = .27, *t*(346) = 4.93, *p <* .001, accounting for 38% of the variance in IGD scores, *R*2 = .38, *F*(1,346) = 24.25, *p <* .001. In other words, for every unit increase in the ADHD score, 8.9 units increase in the IGD score is predicted. Within the females group, the ADHD scale score was a significant predictor of IGD, *β* = .49, *t*(346) = 24.37, *p <* .001, accounting for 23.7% of the variance in IGD scores. In other words, for every unit increase in the ADHD score, 14.4 units increase in the IGD score is predicted. Within the males group, the ADHD scale score was a significant predictor of IGD, *β* = .21, *t*(346) = 4.29, *p <* .001, accounting for 4.2% of the variance in IGD scores, *F*(1,346) = 10.39, *p <* .001. In other words, for every unit increase in the ADHD score, 6.820 units increase in the IGD score is predicted.

### Mediation analysis

A theoretical framework is established based on the previous findings suggesting a potential mediating role of GD in the relationship between ADHD symptoms and academic performance. The mediation model posits that ADHD symptoms may contribute to an increased risk of GD, which in turn could adversely affect academic performance. To test this model, structural equation modeling (SEM) was employed using AMOS software, which allows for the examination of complex relationships between observed and latent variables. This approach is particularly suited to mediation analysis as it enables the estimation of direct and indirect effects while accounting for measurement error and the interrelations among variables. The following steps were taken to ensure the integrity of the mediation analysis: confirming the measurement model for construct validity, and assessing the assumption of multivariate normality. The results of this analysis will provide insights into the mechanistic roles that IGD may play between ADHD symptoms and academic achievement, contributing to a nuanced understanding of how these variables interact within the student population.

The *χ*2 to *df* ratio was 1.786 with *p* < .001 which indicated that the model was an adequate fit Carmines and McIver (1981). The Root Mean Square Error of Approximation (RMSEA) was.049 which indicated a good model fit because it was less than.050. Because the computed PCLOSE (0.600), which tests the null hypothesis that RMSEA is no greater than.05, was significantly greater than.05, there was no evidence to reject the null hypothesis. Additionally, the normed fix index (NFI), the comparative fix index (CFI), and the Tucker-Lewis coefficient (TLI) were.873,.938, and.911, respectively, and suggested that the model fitted very well. The SRMR (the standardized RMR, root mean square residual), which was.038, indicating a very good fit because it was less than.05.

## Discussion

### Gaming disorder prevalence and gender disparities

The findings of this study contribute to the growing body of literature on the prevalence and characteristics of GD and its association with ADHD [12]. The observed prevalence rate of GD in our sample (4.3%) is consistent with rates reported in other studies, suggesting that while GD is not widespread, it is a significant issue among certain subpopulations, particularly in academic settings. The pronounced gender disparity in people with IGD, with males being disproportionately affected, aligns with previous research indicating that gaming disorders are more prevalent among males [24, 36–38]. This may reflect gender differences in gaming habits, socialization patterns, or susceptibility to addiction. The high percentage of males in the DGG underscores the need for gender-specific prevention and intervention strategies.

### Comorbidity of ADHD and GD: Age and gender considerations

The similar mean ages for the ADHD and non-ADHD groups, as well as the DGG and non-DGG groups, suggest that age may not be a differentiating factor for these conditions within the young adult population studied. However, the higher prevalence of GD in the ADHD group compared to the non-ADHD group indicates a potential comorbidity between ADHD and gaming-related problems, which is supported by the substantial overlap in ADHD diagnoses within the GD group [24]. This comorbidity is critical for clinicians and educators to consider when assessing and treating students with either condition. The lack of significant gender differences in ADHD scores challenges some stereotypes that ADHD is more disruptive or prevalent in one gender over the other in the context of higher education. It suggests that the impact of ADHD on individuals may be more related to personal or environmental factors rather than gender alone.

### Impact of gaming habits and ADHD on academic performance

The correlation between GD and gaming hours, particularly on non-university days, suggests that free time may exacerbate gaming behaviors, potentially leading to disorder [39]. This finding has practical implications for the management of GD, as it highlights the importance of structured time and alternative leisure activities as part of the therapeutic approach. The small negative relationship between GD and sleep hours, while expected, is nonetheless significant because it quantifies the sleep disruption associated with excessive gaming [29, 40–42]. The high incidence of nocturnal awakenings in the DGG? to continue gaming is a concerning trend that warrants attention due to the critical role of sleep-in cognitive function, emotional regulation, and overall health [18]. The absence of GD and ADHD symptoms among high academic performers suggests that academic engagement and success may be protective factors against these conditions. Alternatively, it may be that the symptoms of these disorders disrupt academic performance, which would be consistent with the negative correlation observed between disorder prevalence and academic excellence. The correlation analysis indicates a significant positive relationship between GD and ADHD scores (*r* = .265), suggesting a moderate association where increased ADHD symptoms correspond with increased GD symptoms. This is consistent with literature indicating a connection between ADHD and problematic gaming, potentially due to shared attributes like impulsivity and reward-seeking. The stronger correlation for females (*r* = .49) versus males (*r* = .21) suggests a more pronounced ADHD-GD link in females. However, the limited female sample size in the DGG calls for cautious interpretation and underlines the need for research with more female participants. The proportion of ADHD individuals displaying GD symptoms (93.5%) underscores potential comorbidity. The smaller percentage of ADHD without GD (6.5%) suggests variability in the ADHD-GD risk profile, important for clinical ADHD screening. The negative correlation between GD/ADHD and academic excellence (*r* = -.174 and *r* = -.123) indicates potential adverse effects on academic performance, with high performers showing an absence of GD and ADHD symptoms.

### ADHD symptom predictivity on GD severity

The regression analysis provides evidence that ADHD symptoms are a significant predictor of GD severity, with the ADHD scale score explaining a substantial portion of the variance in GD scores (38%). This predictive relationship is quantified by the regression coefficient (*β* = .27), which implies a notable increase in GD scores with each increment in ADHD symptoms. The strength of this relationship is statistically significant and suggests that ADHD traits may exacerbate or contribute to the development of IGD. The gender-specific analysis reveals a more robust predictive value of ADHD for GD among females (*β* = .49) than males (*β* = = .21). This finding is particularly compelling, indicating that ADHD’s impact on GD is more pronounced in females, which aligns with previous research suggesting gender differences in the expression and impact of ADHD. For females, the ADHD score accounts for 23.7% of the variance in GD scores, suggesting that other factors may also play a significant role in GD development in this group. For males, although ADHD is still a significant predictor, it accounts for a smaller percentage of the variance (4.2%), indicating that the relationship between ADHD and GD may be influenced by additional variables not captured in this model.

### Mediating role of GD between ADHD and academic performance

The mediation analysis conducted using SEM provides a robust statistical framework to understand the role of GD as a mediator between ADHD symptoms and academic performance. The results suggest that ADHD symptoms may not only directly impact academic performance but also do so indirectly by increasing the risk of GD. The model fit indices (*χ*2/*df*, RMSEA, PCLOSE, NFI, CFI, TLI, and SRMR) all indicate a good to very good fit, which supports the hypothesized mediation model’s validity. The *χ*2/*df* ratio being below the threshold of 3 suggests that the model is a reasonable approximation of the real data. The RMSEA value being well below the.05 mark and the PCLOSE value being above.05 further reinforce the model’s adequacy. High values of CFI and TLI, along with a low SRMR, indicate that the model’s specified relationships are consistent with the observed data.

## Limitations and future work

The authors recognize several limitations and areas for future research. The cross-sectional design precludes causal inferences, and the convenience sampling method may limit the generalizability of the findings. There is a clear need for longitudinal studies to clarify the causal relationships between ADHD and GD and to track how these relationships evolve over time. Such studies could significantly advance the field by providing evidence for the temporal precedence of one disorder over the other and the directionality of the relationships between ADHD, GD, and academic performance. Manipulating gaming behavior in a controlled setting could yield insights into the causative effects of gaming on ADHD symptoms and vice versa.

The gender imbalance in our study sample, with a significant overrepresentation of males, and the potential cultural and gender inequality influences that may affect females’ disclosure of gaming habits, are acknowledged as key limitations that could skew the data. The concern regarding the low female sample size possibly affecting the observed stronger link between ADHD and Gaming Disorder (ADHD-GD) in females suggests a need for future studies to include a more balanced and larger sample size to ensure more accurate and generalizable findings. Additionally, the limitation of our study being conducted at a single university site calls for future research to encompass multiple geographical locations to enhance the robustness and applicability of the results. Exploring the differences in gaming habits between individuals with ADHD who are treated versus untreated, as well as the co-occurrence of gaming disorder with other drug or behavioral addictions, are proposed as valuable directions for future work to provide a more comprehensive understanding of gaming disorder and its multifaceted nature.

Future studies should consider the bifactors of the ASRS v1.1 in more details, as delineated by Stanton et al., to deepen our understanding of ADHD’s complexity and its relationship with Gaming Disorder (GD). Such an approach could unveil nuanced insights into the differential impact of these ADHD dimensions on GD, potentially leading to more targeted intervention strategies.

## Conclusion

This research study adds to the literature and provides evidence of a significant positive relationship between GD and ADHD symptoms, with gender-specific variations indicating a stronger correlation in females than males. The study also uncovers a negative association between these disorders and academic performance, particularly in high-performing students, suggesting that GD and ADHD symptoms may be potential barriers to academic success.

The mediation analysis using structural equation modeling (SEM) further elucidates the role of GD as a mediator in the relationship between ADHD symptoms and academic performance, offering insights into the complex interplay of these factors in university students. The findings have broad implications for the theoretical understanding of ADHD and GD comorbidity and the necessity for screening of both disorders in affected populations. They also underscore the importance of recognizing and addressing the interconnectedness of behavioral disorders and academic performance in the university student population.
